# Identification of the two-component guaiacol demethylase system from *Rhodococcus rhodochrous* and expression in *Pseudomonas putida* EM42 for guaiacol assimilation

**DOI:** 10.1186/s13568-019-0759-8

**Published:** 2019-03-11

**Authors:** Javier García-Hidalgo, Krithika Ravi, Lise-Lotte Kuré, Gunnar Lidén, Marie Gorwa-Grauslund

**Affiliations:** 1 Division of Applied Microbiology, Department of Chemistry, Lund University, P.O. Box 124, 221 00 Lund, Sweden; 2 Department of Chemical Engineering, Lund University, P.O. Box 124, 221 00 Lund, Sweden

**Keywords:** Guaiacol demethylase, Cytochrome P450, *Rhodococcus rhodochrous*, *Pseudomonas putida*, Lignin, Aromatic compound catabolism

## Abstract

A diversity of softwood lignin depolymerization processes yield guaiacol as the main low molecular weight product. This key aromatic compound can be utilized as a carbon source by several microbial species, most of which are Gram positive bacteria. Microbial degradation of guaiacol is known to proceed initially via demethylation to catechol, and this reaction is catalyzed by cytochrome P450 monooxygenases. These enzymes typically require a set of redox partner proteins, whose number and identities were not described until very recently in the case of guaiacol. In this work we identified two proteins involved in guaiacol demethylation by the actinomycete *Rhodococcus rhodochrous*. Additionally, we constructed four different polycistronic operons carrying combinations of putative redox partners of this guaiacol demethylation system in an inducible expression plasmid that was introduced into the Gram negative host *Pseudomonas putida* EM42, and the guaiacol consumption dynamics of each resulting strain were analyzed. All the polycistronic operons, expressing a cytochrome P450 together with a putative ferredoxin reductase from *R. rhodochrous* and putative ferredoxins from *R. rhodochrous* or *Amycolatopsis* ATCC 39116 enabled *P. putida* EM42 to metabolize and grow on guaiacol as the sole carbon source.

**Electronic supplementary material:**

The online version of this article (10.1186/s13568-019-0759-8) contains supplementary material, which is available to authorized users.

## Introduction

The aromatic compound guaiacol (2-methoxyphenol) is one of the main low-molecular weight products obtained after alkaline depolymerization of softwood lignins (Pandey and Kim 2011; Gosselink et al. 2012; Abdelaziz et al. 2018), and it is also found with other feedstocks and depolymerization methods. For this reason, guaiacol assimilation has recently been identified as a key target to increase the efficiency of microbial lignin valorization (Beckham et al. 2016). Guaiacol is a difficult and non-preferred substrate that is toxic to some bacterial species (Vicuña et al. 1987; Chow et al. 1999). Nevertheless, its bacterial degradation has been described both in Gram positive and negative bacteria, including several anaerobic species (Kofli et al. 2005; Studenik et al. 2012). This bacterial degradation proceeds through the *O*-demethylation of guaiacol in one single step to generate catechol (1,2-dihydroxybenzene). In a few cases, namely *Moraxella* sp. GU2 (Dardas et al. 1985), *Rhodococcus rhodochrous* 116 (Eltis et al. 1993; Karlson et al. 1993) and *Streptomyces setonii* 75Vi2 (currently *Amycolatopsis* sp. ATCC 39116) (Sutherland 1986), the enzymes responsible for the aerobic demethylation of guaiacol have been identified as soluble cytochrome P450 monooxygenases.

**Fig. 1:**
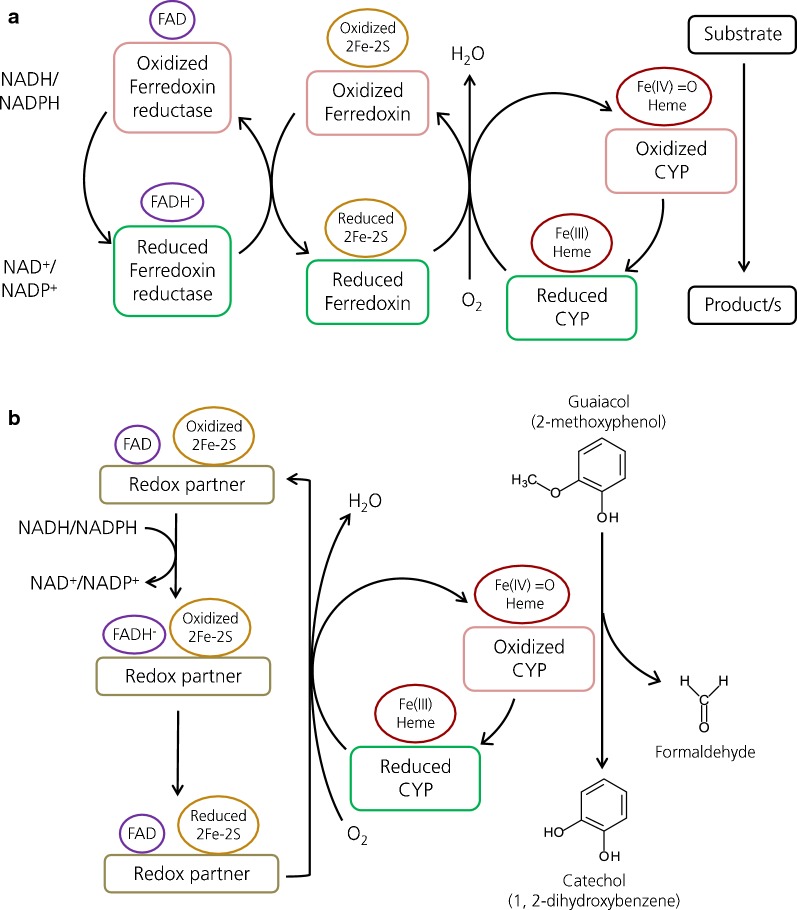
**a** General diagram of three-component CYP systems comprised of a CYP monooxygenase and two redox partners, namely ferredoxin and ferredoxin reductase. Prosthetic groups on each protein as well as their redox state are depicted. **b** Diagram of the reaction carried out by the guaiacol *O*-demethylation system of *Rhodococcus rhodochrous* and the electron transport involved between the two components

Cytochrome P450s (CYPs) are a broadly distributed family of enzymes with a huge range of activities and functions. These enzymes usually rely on redox partner proteins for electron transfer from a cofactor such as NAD(P)H to the heme group in the CYP enzyme. Most of the bacterial soluble CYPs belong to class I (Kelly and Kelly 2013), where the electron transport is usually achieved by two redox partner proteins (Katagiri et al. 1968; Kawahara et al. 1999; Chun et al. 2007; Tripathi et al. 2013): the first element is an FAD-containing ferredoxin reductase able to interact with the redox cofactor, and the second protein is an iron–sulfur cluster-containing ferredoxin, which can transfer the electrons from the reductase to the CYP (Fig. 1a).

Very recently, the guaiacol demethylation system from *Amycolatopsis* sp. ATCC 39116 has been described in detail, showing a novel arrangement where only one redox partner protein, named GcoB, was shown to carry out the function of electron transfer from NADH to the CYP monooxygenase GcoA (Mallinson et al. 2018). This system from *Amycolatopsis* sp. ATCC 39116 has been proven to represent a novel class of soluble CYP enzymes. Homologous genes from this and other actinomycete species (*Rhodococcus jostii* RHA1 and *R. pyridinivorans* AK37) were heterologously expressed in *Pseudomonas putida* KT2440 using a plasmid-based system (Tumen-Velasquez et al. 2018). However, the results with the *Rhodococcus* strains were not as successful as in the case of *Amycolatopsis*, and no growth was reported with the genes from rhodococci using guaiacol as sole carbon source.

Among rhodococci, the identity of the CYP responsible for guaiacol demethylation in *R. rhodochrous* was the first one to be described in a Gram positive bacterium (Eltis et al. 1993; Karlson et al. 1993). However, no identification or further characterization of the necessary redox partners involved in this environmentally relevant activity were reported. In light of this knowledge gap and due to the poor results obtained with other *Rhodococcus* species, we decided to have a deeper look into the guaiacol demethylation system in *R. rhodochrous*. In the present work we aimed at identifying the proteins responsible for guaiacol demethylation in *R. rhodochrous* strain J3, with the additional purpose of implementing this system in the streamlined bacterial host *P. putida* EM42 (Martínez-García et al. 2014). The *P. putida* EM42 strain, just like its parental strain KT2440, is able to utilize catechol but not guaiacol as a carbon source. This fact makes *P. putida* an ideal host to introduce this demethylation step that converts guaiacol into catechol, and would potentially allow this host bacterium to metabolize guaiacol, adding this lignin-related compound to the ample repertoire of aromatic carbon sources that *P. putida* can naturally process.

## Materials and methods

### Chemicals

Restriction enzymes and T4 DNA ligase were obtained from Thermo Fisher Scientific (Vilnius, Lithuania). DNA oligonucleotides were synthesized by Eurofins genomics (Ebersberg, Germany). All other reagents were purchased from Sigma-Aldrich (St. Louis, USA).

The five genes used to generate the different combinations of elements were synthesized by Thermo Fisher GeneArt gene synthesis service (Regensburg, Germany), with a specific codon optimization for *P. putida* performed by the manufacturer as part of the GeneOptimizer process. Optimized Ribosome Binding Sites (RBS) and immediately downstream sequence (AGGAGGAAAAACAT) were added upstream of every gene to be expressed (Silva-Rocha et al. 2013). GenBank accession numbers for the nucleotide sequences of synthetized genes (including RBS and restriction sites) are as follows: CYP gene MK007067, redox partner gene MK007068, ferredoxin 1 gene from *R. rhodochrous* MK007069, ferredoxin 2 gene from *R. rhodochrous* MK007070 and ferredoxin gene from *Amycolatopsis* MK007071.

Accession numbers for the five native nucleotide sequences used for gene synthesis as well as synthetic nucleotide sequences for each one of the genes are shown in Additional file 1. Accession numbers for the amino acid sequences of each protein are shown in Table 1.

**Table 1:** *P. putida* EM42 strains used in this study, their corresponding plasmids and cloned elements thereof

Strain name	Plasmid	Cytochrome P450	Ferredoxin reductase	Ferredoxin
Control	pSEVA424	None	None	None
G0	pSEVA424_P450Rrh	WP_085469912 from *R. rhodochrous* J3	None	None
GI	pSEVA424_P450Rrh_FrdRrh_FdxRrh1		WP_085469913 from *R. rhodochrous* J3	WP_085470952 from *R. rhodochrous* J3
GII	pSEVA424_P450Rrh_FrdRrh_FdxRrh2			WP_085469096 from *R. rhodochrous* J3
GIII	pSEVA424_P450Rrh_FrdRrh_FdxAmy			WP_020416430 from *Amycolatopsis* ATCC 39116
GIV	pSEVA424_P450Rrh_FrdRrh			None

### Bacterial strains and plasmids

*Escherichia coli* TOP10 (F-*mcr*A Δ(*mrr*-*hsd*RMS-*mcr*BC) φ80*lac*ZΔM15 Δ*lac*X74 *rec*A1 *ara*D139 Δ(*ara*-*leu*)7697 *gal*U *gal*K *rps*L (Str^R^) *end*A1 *nup*G) was used for routine cloning experiments, *P. putida* EM42 (Martínez-García et al. 2014) was used for the expression of the plasmid constructs in guaiacol assimilation experiments.

The plasmid pSEVA424 used as scaffold in this work was obtained from the Standard European Vector Architecture (SEVA) repository (Silva-Rocha et al. 2013). All plasmids used or constructed in this study are listed in Table 1, with the corresponding *P. putida* EM42 resulting strains.

### Media and culture conditions

LB medium (tryptone 10 g/L, sodium chloride 10 g/L and yeast extract 5 g/L) was used for routine growth of *E. coli* and *P. putida* during the cloning experiments. Mineral M9 medium (Sambrook 2001) with 1% (v/v) trace element solution from Pfennig and Lippert (1966) was employed for all the guaiacol assimilation experiments and for growth of *P. putida* pre-inoculum. Spectinomycin 100 µg/mL and IPTG 1 mM were added from the start to the pre-inoculum and guaiacol experiments in M9 medium. Stock solutions of guaiacol 10× (50 mM) and d-glucose 10× (100 g/L) were sterilized through a 0.2 µm filter prior to their addition to the corresponding media.

Pre-inoculum of all *P. putida* strains were cultured overnight in M9 medium with 10 g/L glucose at 30 °C with orbital shaking at 180 rpm. Guaiacol assimilation experiments were carried out in 250 mL shake flasks with 25 mL of M9 medium with spectinomycin 100 µg/mL and IPTG 1 mM in the same conditions described for the pre-inoculum with guaiacol 5 mM and with or without glucose 10 g/L. Biological duplicates were done for all the experiments. Aliquots were withdrawn regularly for measurement of optical density at 620 nm (OD_620_) and HPLC analysis of the concentration of guaiacol and other intermediates.

### Construction of CYP-encoding plasmids

**Fig. 2:**
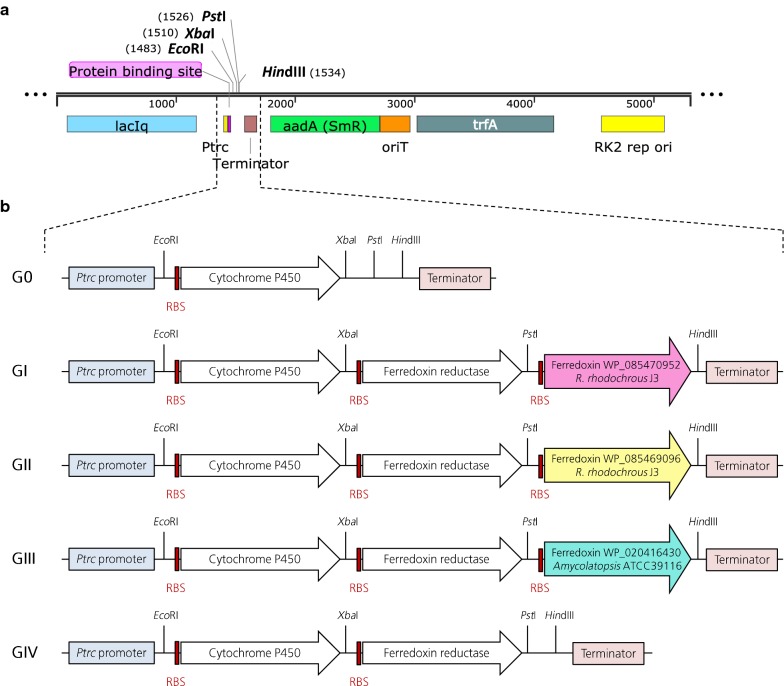
**a** Linear map of pSEVA424 plasmid and its relevant features. **b** Structure of the polycistronic operons constructed in pSEVA424. Strains GI, GII, and GIII contain all three gene elements, strain G0 only carries the first gene (CYP gene), and strain GIV contains the first two genes only. Strains GI–III differ by the origin of the ferredoxin gene (WP_085470952 from *R. rhodochrous* J3 in GI, WP_085469096 from *R. rhodochrous* J3 in GII and WP_020416430 from *Amycolatopsis* ATCC 39116 in GIII)

Each one of the synthetic genes was digested with the two corresponding restriction enzymes as indicated in Fig. 2b. Subsequently, the digested fragments were assembled into pSEVA424 (Fig. 2a) and the constructions were verified by restriction analysis and Sanger sequencing to verify the absence of mutations (Eurofins genomics, Ebersberg, Germany). Resulting plasmids were used to transform electrocompetent *P. putida* EM42 cells using 0.2 cm gap width cuvettes with a Gene Pulser apparatus equipped with a Pulse Controller (Bio-Rad, Hercules, USA) following the electroporation protocol from Martínez-García and de Lorenzo (2012). The strains obtained with each one of the different plasmids are listed in Table 1.

### Chromatographic analysis

Prior to the analysis, all the culture aliquots were centrifuged at 20,000×*g* for 3 min to remove the cells, and then the supernatants were filtered through a 0.2 µm filter to remove any possible aggregates in suspension. Analysis of the concentration of guaiacol and aromatic intermediates was performed with a Waters Acquity HPLC system (Milford, USA) coupled with a UV detector. The column used was an Agilent InfinityLab Poroshell 120 EC-C18 with an internal diameter of 4.6 mm, 100 mm length and 4 μm particle size. Temperature of the column was maintained at 50 °C. The mobile phases used were the binary solvent system consisting of fraction A (99.5% water and 0.5% acetic acid) and fraction B (99.5% acetonitrile and 0.5% water). The injected sample volume was 5 μL. A gradient elution method with a flowrate of 1 mL/min was used for analysis. The method started with 97% A, decreased to 85% A in 12 min, held at 85% A for 3 min and decreased to 20% A in 5 min. After every sample, the column was washed with 90% B for 5 min and equilibrated with 97% A for 10 min.

Glucose was analyzed on an HPLC coupled with an RI detector (Waters, Milford, MA, USA). An Aminex HPX-87H (Bio-Rad, Hercules, USA) column (60 °C) with 0.6 mL/min flow rate of 5 mM sulfuric acid as mobile phase was used. The injection volume was 20 μL with a run time of 50 min for each sample.

Resulting data was reviewed with Empower 3 Chromatography Data Software. Peaks were quantified according to the area under the curve against their authentic calibration standards.

## Results

### Identification of putative genetic elements of *R. rhodochrous* J3 demethylase system

**Fig. 3:**
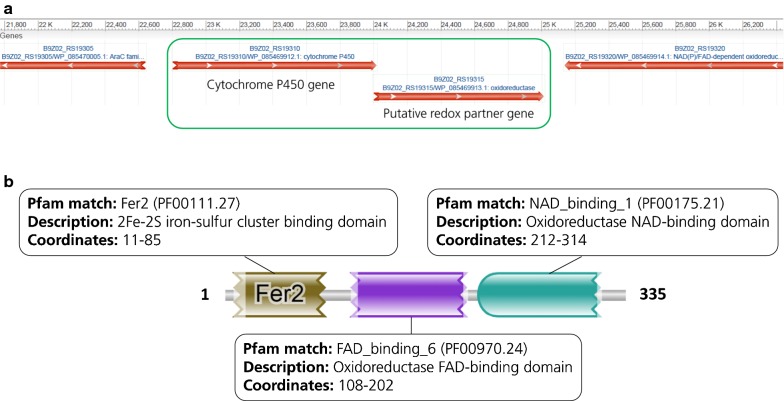
**a** Detail of the local genomic context of the CYP gene in *Rhodococcus rhodochrous* J3 and its companion gene encoding a redox partner protein. **b** Results of HMMER analysis of the amino acid sequence of the redox partner from *Rhodococcus rhodochrous* J3 (WP_085469913.1), showing three predicted domains

The gene encoding the CYP responsible for the conversion of guaiacol into catechol in *R. rhodochrous* J3 was found with the help of a partial amino acid sequence reported 25 years ago as part of the characterization of this monooxygenase enzyme. The reported 21 amino acid-long N-terminal partial sequence TSTLSWLDEITMEELERNPYP (Eltis et al. 1993), was used to identify the corresponding gene in the genome of *R. rhodochrous* J3 (taxon ID 903528, accession number NZ_FXAV01000012.1), finding a 100% identity with the CYP protein encoded by a specific gene (locus tag B9Z02_RS19310). After examination of the genomic context of this gene (Fig. 3a), only one companion gene could be found immediately downstream (locus tag B9Z02_RS19315), that seemed to constitute an operon with the CYP gene, in a similar fashion as observed in other CYP systems (van Beilen et al. 2005; Chun et al. 2007); however, the potential third ferredoxin element of this system seemed to be missing or located in a different genomic position. The product of this companion gene was analyzed with the HMMER tool (Finn et al. 2015) in order to predict conserved functional domains in this protein, showing the three-domain arrangement shown in Fig. 3b.

In order to identify a putative ferredoxin element in *R. rhodochrous* J3, two different ferredoxin amino acid sequences were aligned using the BLASTP suite with this genome and other complete genomes from guaiacol-degrading bacteria, among them *Amycolatopsis* sp. ATCC 39116 that is also known to degrade guaiacol. The protein sequences chosen for this search were WP_014075176 from *Sphingobium* sp. SYK-6, a ferredoxin known as LigXc and reported to be involved in the demethylation of another guaiacyl lignin compound (Yoshikata et al. 2014), and BAK65525 from *Mycobacterium* sp. HXN-1500, which belongs to a well-characterized CYP system from a Gram positive bacterium (van Beilen et al. 2005). These two sequences share an identity of only 36% with each other, however genomic searches using both of these sequences independently led to the same two top hits in *R. rhodochrous* J3 (accession numbers WP_085470952 and WP_085469096) and to one single candidate in the genome of *Amycolatopsis* sp. ATCC 39116 (accession number WP_020416430). In light of this result we decided to test these three proteins as putative ferredoxins. In order to provide a clear picture of the similarity of the proteins used in this study, those used in Tumen-Velasquez et al. (2018) and other homologous proteins from guaiacol-degrading actinomycetes, an amino acid identity matrix was built for each component (Additional file 2). It clearly indicates that the demethylation system from *R. rhodochrous* J3 is almost identical to that from *R. pyridinivorans* AK37, which did not previously enable *P. putida* KT2440 to degrade guaiacol as reported in Tumen-Velasquez et al. (2018).

### Evaluation of various demethylase system combinations in *P. putida*

The selected genes were codon-optimized and their synthesis was outsourced. These genes were assembled in polycistronic operons into the pSEVA424 scaffold as illustrated in Fig. 2b, and the *P. putida* EM42 host was transformed with these plasmids giving rise to strains G0 to GIV (Table 1).

The first negative control of these experiments was a *P. putida* EM42 strain harboring the empty expression plasmid pSEVA424. The second control was called strain G0, and it contained only the CYP gene without any redox partner. Strains GI, GII and GIII carried the same CYP gene from *R. rhodochrous* J3 followed by the native companion gene encoding a putative ferredoxin reductase together with one of the three aforementioned ferredoxin candidates. Finally, strain GIV contained the CYP gene followed only by the putative ferredoxin reductase gene, in a configuration that mimicked the native operon without any ferredoxin element.

**Fig. 4:**
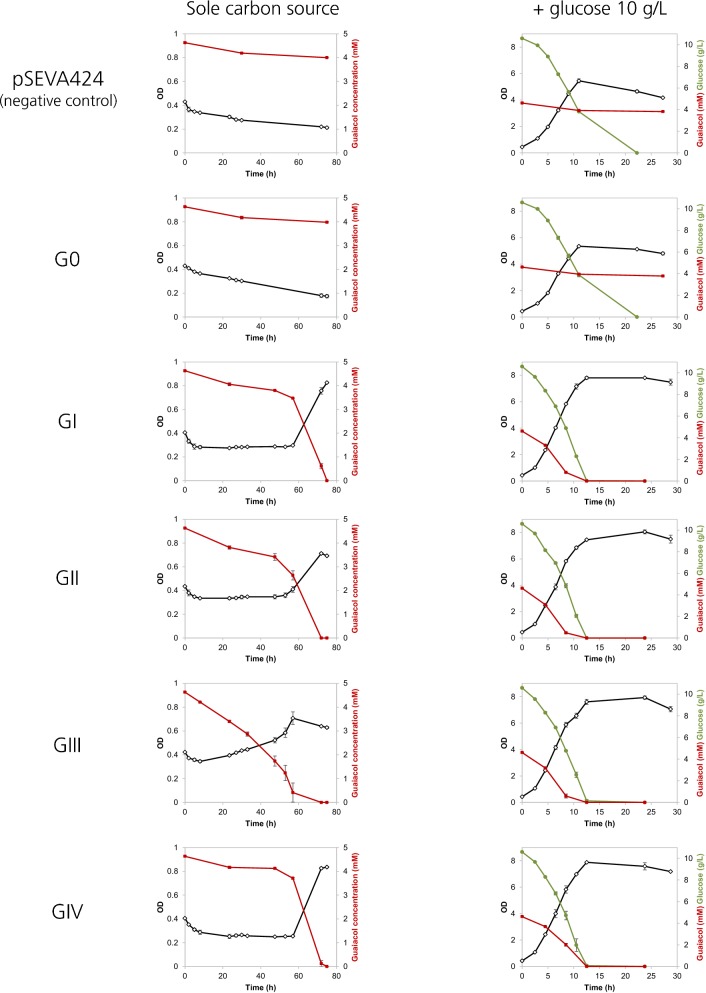
Guaiacol assimilation experiments with the six strains used in this study in M9 mineral medium with guaiacol 5 mM as sole carbon source (left panels) and in the presence of glucose 10 g/L (right panels). OD_620_ is shown in black, guaiacol concentration is shown in red and glucose concentration is shown in green. Vertical error bars represent standard deviation

The ability of these 6 constructed strains to consume guaiacol was tested in liquid mineral medium M9 at 30 °C with orbital shaking, with guaiacol 5 mM (0.62 g/L) as sole carbon source or in the presence of glucose 10 g/L. Spectinomycin 100 µg/mL was present in all cases to avoid plasmid loss and IPTG 1 mM was added to induce the expression of the synthetic operons. Growth was measured by monitoring OD_620_, and the concentration of guaiacol was assessed by HPLC (Fig. 4). The protein expression pattern of each constructed strain was analyzed by SDS-PAGE to rule out possible hyperexpression or accumulation of any of the gene products under induction conditions (Additional file 3). No evident differences in the expression pattern were detected in any strain compared to the negative control.

None of the negative controls (pSEVA424 and G0) were able to consume guaiacol (Fig. 4). In contrast, all the strains expressing polycistronic constructions (GI, GII, GIII and GIV) were able to consume guaiacol as the sole carbon source, leading to its complete disappearance in a time frame ranging from 60 to 72 h. Strains GI, GII and GIV assimilated guaiacol as sole carbon source at a very similar rate, whereas guaiacol was completely consumed by strain GIII at an earlier time point than for the other strains in these conditions. Regarding growth, there was clear biomass production and the OD curves of strains GI to GIV were noticeable different to those of the strains pSEVA424 and G0 (Fig. 4). The biomass production came after an initial drop in OD_620_, most likely attributable to the change of conditions with respect to the pre-inoculum on glucose. Strains GI, GII, GIII and GIV were also able to consume 5 mM guaiacol in the presence of glucose in approximately 10 h as shown in Fig. 4. At the end of cultivations with glucose the final OD is higher in strains GI–GIV, indicating overall increased biomass with respect to the controls pSEVA424 and G0.

To assess the possible toxic effect of guaiacol consumption on *P. putida* EM42 viability, we also performed endpoint CFU/mL measurements of each strain with and without glucose. These measurements indicated no decrease in viability of the host cells after guaiacol consumption (Additional file 4).

## Discussion

In this study we identified the operon involved in guaiacol demethylation in one of the first bacteria reported to be able to use this aromatic compound as sole source of carbon and energy, *Rhodococcus rhodochrous* (Eltis et al. 1993). Furthermore, the guaiacol demethylation activity was successfully implemented into *Pseudomonas putida* EM42—a strain derived from *P. putida* KT2440, a bacterial host that has become one of the platform organisms of choice to carry out harsh and demanding chemical transformations (Nikel and de Lorenzo 2018). It was found that the cytochrome P450 monooxygenase gene was located upstream of a gene encoding an oxidoreductase, pointing to its probable role as a redox partner for the CYP. This was confirmed when the combined expression of the two genes enabled *P. putida* to metabolize guaiacol. All the strains harboring both genes were able to completely consume and grow on guaiacol as sole carbon source within about 70 h and the ability to consume guaiacol was maintained in the presence of glucose. The obtained results show that the oxidoreductase gene was sufficient in addition to the CYP gene to perform the demethylation of guaiacol in the transformed *P. putida* strain, and suggests that this is likely the case also in *R. rhodochrous.* This would be in agreement with recently reported data for the *Amycolatopsis* sp. ATCC 39116 system (Tumen-Velasquez et al. 2018).

This ability to demethylate guaiacol without the need of a ferredoxin partner differs from most of the bacterial CYP systems described so far that present a three-component electron transport system comprising a CYP, a ferredoxin and a ferredoxin reductase (Guengerich and Munro 2013; Kelly and Kelly 2013) (Fig. 1a). The expression of these three elements is in some cases coordinated in an operon, such is foe example the case of the alkane hydroxylase from *Mycobacterium* sp. HXN-1500 (van Beilen et al. 2005); in other cases however, the different components are found at different locations in the genome, for instance in the camphor hydroxylase system from *P. putida* (Katagiri et al. 1968) or the fatty acid hydroxylase from *Streptomyces coelicolor* A3(2) (Chun et al. 2007). This made us consider the possibility of a third protein in a different locus of the genome that could strengthen the *R. rhodochrous* demethylase system. However, overexpression of putative ferredoxin genes from *R. rhodochrous* J3 and *Amycolatopsis* sp. ATCC 39116 genomes did not further improve guaiacol consumption in *P. putida*, indicating that the two-component system was self-sufficient. We hypothesize that the oxidoreductase gene from *R. rhodochrous* must encode a hybrid protein containing an iron–sulfur cluster, an FAD prosthetic group and must be able to interact with NAD(P)H and with the CYP enzyme. In fact, when the sequence of this redox partner gene was analyzed with the HMMER tool from EMBL-EBI three domains were recognized, namely a 2Fe–2S iron–sulfur cluster on the N-terminal region followed by a central FAD-binding domain and an NAD-binding domain in the C-terminus (Fig. 3b). These results coincide with the recent findings reported in Tumen-Velasquez et al. (2018) and Mallinson et al. (2018), confirming the existence of a novel class of two-element CYP system responsible for the demethylation of several *O*-methylated aromatic compounds in actinobacteria (Fig. 1b). This two-component CYP system seems to be the rule rather than an exception for *O*-demethylation of methoxylated aromatic compounds in actinomycetes (and also probably ethoxylated, as demonstrated in Mallinson et al. (2018) with guaethol).

In parallel to the identification of the demethylase system, we reached our second goal which was to convey the ability to use guaiacol to a bacterial host that has become one of the platform organisms of choice to carry out harsh and demanding chemical transformations, *P. putida* KT2440 and its derived strains (Nikel and de Lorenzo 2018).

The developed plasmid-based system enables *P. putida* to convert guaiacol into catechol, that can be further metabolized by this bacterium into central metabolism intermediates and formaldehyde (Fig. 1b) that is quickly converted into formic acid and methanol in a detoxification process carried out by a dismutase enzyme (Adroer et al. 1990). The intracellular release of highly reactive formaldehyde due to the assimilation of guaiacol does not seem to pose a problem for *P. putida* EM42. This is not surprising, since a similar *O*-demethylation reaction is carried out by VanAB in the parental *P. putida* KT2440 for the conversion of vanillic acid into protocatechuate, entailing a quick production of formaldehyde. On the contrary, it is noteworthy that LigM, the enzyme carrying out the same reaction in *Sphingobium* sp. SYK-6 is THF-dependent and unlike VanAB does not release formaldehyde (Kamimura et al. 2017). The latter mode of conversion might be more suitable for its implementation in bacteria that cannot tolerate formaldehyde as robustly as *P. putida* KT2440.

In contrast with the recent work with *R. pyridinivorans* and *R. jostii* genes (Tumen-Velasquez et al. 2018), those from *R. rhodochrous* not only allowed *P. putida* to consume guaiacol, but also supported growth on guaiacol as a sole carbon source.

Successful plasmid expression of the *R. rhodochrous* system in *P. putida* EM42 might be explained by several factors. First, the vector chosen for the construction of this system was the expression plasmid pSEVA424 (Martínez-García et al. 2015), that presents the broad host-range origin of replication RK2, leading to a copy number that can be as low as two plasmids per chromosome (Jahn et al. 2016), making these results more easily comparable with single copy genomic insertions. Also, the expression system harbored in pSEVA424 is *lacI*^q^/*Ptrc*, a system oriented to its application in metabolic engineering. It presents an IPTG-induced hybrid promoter (*Ptrc*), yielding a relatively high level of transcription and homogeneous populations (Balzer et al. 2013). In addition, artificially synthesized genes with an optimized codon usage for *P. putida* were used, avoiding transcription problems due to rare codons; this was of particular importance considering that we aimed to clone genes from a high G+C content Gram positive bacterium into a Gram negative host. Each individual gene was also preceded by an optimized RBS and spacer sequence, which also contributes to standardize the level of expression of each cistron (Silva-Rocha et al. 2013), and further increases the translation efficiency of the system.

Finally, another key element of this system was the host itself, *P. putida* strain EM42. This platform strain is the result of a deep genome editing of the parental KT2440 strain in order to achieve higher ATP and NAD(P)H availability, increased genetic stability and metabolic robustness (Martínez-García et al. 2014). All these features may have made it even more suitable than the parental strain for the functional expression of heterologous metabolic pathways with high demands of reducing power, such as the assimilation of guaiacol in our case. Streamlined strains, such as EM42, will in the near future be important tools for the implementation of new exogenous pathways enabling the metabolism of more aromatic compounds by *P. putida* and thereby improving the valorization of depolymerized lignin.

## Additional files

**Additional file 1.** Codon-optimized nucleotide sequences.

**Additional file 2.** Amino acid identity matrix of components from guaiacol demethylation systems.

**Additional file 3.** SDS-PAGE analysis of protein expression pattern of *P. putida* EM42 strains.

**Additional file 4.** CFU/mL measurement of the recombinant strains used in this study.

## Abbreviations

CYP: cytochrome P450
NAD(P)H: reduced nicotinamide adenine dinucleotide (phosphate)
FAD: Flavin adenine dinucleotide
NAD: nicotinamide adenine dinucleotide
RBS: ribosome binding site
IPTG: isopropyl β-d-1-thiogalactopyranoside
LB: Lysogeny Broth
rpm: revolutions per minute
*g*: gravitational force
OD_620_: optical density at 620 nm wavelength
HPLC: high performance liquid chromatography
CFU: colony-forming units
2Fe–2S: iron–sulfur cluster with 2 iron and 2 sulfide ions
G+C: guanine and cytosine content
