# Supplementary material for: Identification of the two-component guaiacol demethylase system from Rhodococcus rhodochrous and expression in Pseudomonas putida EM42 for guaiacol assimilation
Source: AMB Express. 2019 Mar 11;9:34. doi: 10.1186/s13568-019-0759-8 (PMC6411806; doi:10.1186/s13568-019-0759-8)
Supplement: Supplementary file 1 — Additional file 1. Codon-optimized nucleotide sequences. [file 13568_2019_759_MOESM1_ESM.pdf]

AMB Express

Title

Identification of the two-component guaiacol demethylase system from *Rhodococcus rhodochrous* and expression in *Pseudomonas putida* EM42 for guaiacol assimilation

Authors

Javier García-Hidalgo [a,*], Krithika Ravi [b], Lise-Lotte Kuré [a], Gunnar Lidén [b], Marie Gorwa-Grauslund [a]

[a] Division of Applied Microbiology, Department of Chemistry, Lund University, P.O. Box 124, SE-221 00 Lund, Sweden

[b] Department of Chemical Engineering, Lund University, P.O. Box 124, SE-221 00 Lund, Sweden

* Corresponding author: javier.garcia_hidalgo@tmb.lth.se Phone number: +46 462228328

Additional file S1

Codon-optimized nucleotide sequences

The codon-optimized nucleotide sequences of the five synthetic genes used in this work are shown below. Restriction sites for cloning into the vector pSEVA424 are shown in red, ribosome binding sites are marked in blue, start codons are marked in green, stop codons are marked in orange.

Cytochrome P450 from *Rhodococcus rhodochrous* J3 (*Eco*RI-*Xba*I)

GenBank accession number: MK007067

Native sequence: locus tag B9Z02_RS19310

GAATTCAGGAGGAAAAACCCATGACCAGCACCCTGAGCTGGCTGGACGAGATCACCATGGAAGAACTG
GAACGCAACCCGTATCCGGTGTACGAGCGCCTGCGTGCCGAAGCGCCAATCGCCTTCGTGCCAGTGCT
GGGTGCCTACGTGGCCAGCACCACCGAAGCCTGCCGTGCCGTGGCCGCCGGTGACGACTTCGACGGCA
TCATCACCCCAGCCGGTGGCCGTACCTTCGGCCATCCAGCCATCATCGGCGTGAACGGCGACATCCAC
CGCGACCTGCGCAGCATGGTGGAACCAGCGCTGCAGCCAGCCGAGGTGGACCGCTGGATCGAGGATCT
GGTGCGTCCGATCGCGCGTCGCTACGTGGAAGCCTTCGAGGCCGACGGCAAGGCCGATCTGGTGGCCC
AGTTCTGCGAGCCGGTGAGCGTGCGTAGCCTGGGCGATCTGCTGGGCCTGAAGGACGTGAGCAGCGAC
AAGCTGCGCGAGTGGTTCCACAAGCTGAGCGACAGCTTCACCAACGCCGCCATGGACGAGGACGGCAA
CTTCCTGAACCAAGAGCGCTTCGACGAGGGCGACCGTGCCAAAGAAGAGATCCGCAGCGTCGTGGACC
CACTGATCGACCATTGGATCGAGCACCCGGACGACAGCGCCATCAGCCACTGGCTGCACGACGGCATG
CCCGAGGGTCAGACCCGTGACCGCGACTACATCTACCCGACCCTGTACGTGTTCCTGCTGGGTGCCAT
GCAAGAACCGGGTCACGCCATGTCGTCGACCCTGGCCGGTCTGTTCACCCGTCCGGAACAGTTCGAAG
CCGTGGTGGACGAACCCGGTCTGATCCCACGTGCGATCGCCGAGGGCATGCGTTGGACCTCGCCAATC
TGGTCGGGCACCGCGCGTATCGCCAAGCGCGATACCGTGGTGAGCGGCATCGAGATCAGCGAGGGCAG
CGTGGTGATGCTGAGCTACGGCAGCGCCAACCACGACATCGACGTGTTCGATGCCCCAAGCCGCTACG
ATCTGACCCGTCCGCCACTGCCGCACCTGGCCTTCGGTGCCGGTAAACATGCCTGCGCCGGTATCTAC
TTCGCCAACAACGTGAGCCGCATCGGCCTGGAAGAACTGCTGGAAACCATCCCGAACCTGGAACGCGA
CACCAGCGAGGACGTCGAGTTCTGGGGCTGGGGCTTCCGTGGTCCGAAAACCCTGCATGCCCGTTGGG
AGATCTGAATCTAGA

Redox partner protein from *Rhodococcus rhodochrous* J3 (*Xba*I-*Pst*I)

GenBank accession number: MK007068

Native sequence: locus tag B9Z02_RS19315

TCTAGAAGGAGGAAAAACCCATGGGCGACCTGACCATGAGCTACACCCTGACCGCCGGCACCGGTGTG
GTGCCGTGCGAACCGGGTCGTACCGTGCTGGAAGCCTTCCTGCGCAACGGCAACTGGATGCCGAACAG
CTGCAACCAGGGCACCTGCGGTACCTGCAAGATCAAGGTGCTGGACGGCGAGCTGGACCACCGCAACA
GCCCAGAAGCGACCCTGACCGCGGACGAACTGGCCGACGGCTTCGTGCTGGCCTGCCAAGCGACCCCA
CGCGGTGACGTGGTGTTCGAAACCCCAGCCACCGAGGAAAGCGCGGCGACCCACGCGCTGCGCGACGT
GGTCGCCACCGTGACCGAAGTGCGTGATATCGCCGCGGGCACCCGCAAGGTCCTGCTGACCGCCGATG
AGCCGCTGGAATTCAGCGCCGGTCAGTACGTGGAAGTGACCGTGCCAGGCACCGAGATCCGTCGCCAG
TACAGCCTGGCGAACCCACCGTGCGAAACCAAGCAGCTGGAACTGCACATCCGTCGTCAGCCAGGCGG
TGTGGCCAGCGAGTGGGTGTTCGAGCGCATCGACGTGGGCGAACGCGTGGCCGTGACCGGTCCGTACG

GCGACTTCACCTTCGATCCGGAAGGCACCACCCCGATCGCGCTGCTGGGTGGTGGCACCGGTCTGGCC
CCACTGGAAGCCATCGTGCGTCAGGCCCTGTCGCTGGCCCCAGACCGTCAGATCCTGCTGTACCACGG
CGTGCGTACCTGCGCCGACCTGTACGACGTGGATTTCCTGCGCGAACTGGAAACCCGTCATCCGGGTT
TCCGCTACGTGACCTGCGTGAGCCGTGAGAGCGGTGGCGATCGTGCCGGTTACGTGACCGACGCCTTC
CTGGAAGATGTGGCCTCGGCCAAAGAGTTCACCGGCTACATCTGCGGCAGCGAGGCCTTCGTCGAGGC
CAGCGTCAAGGCCTTCAAACGCCGTCGCATGAGCCCACGCCGTATCCGTCGCGAGCGTTTCACCCCAG
CCGGTTGACTGCAG

Ferredoxin 1 from *Rhodococcus rhodochrous* J3 (*Pst*I-*Hind*III)

GenBank accession number: MK007069

Native sequence: locus tag B9Z02_RS27210

CTGCAGAGGAGGAAAAACCCATGCCGACCGTGGTGTACCAGCTGCCGGATGGCACCACCTCGAGCGTG
GATGTGCCAGCCGGTCAGAGCGTGATGGACGGCAGCGTGCGCAACAACCTGCCAGGCATCATCGCCGA
GTGCGGTGGCAGCTGCTCGTGCGCGACCTGCCACGTGTACGTGGACGGTGGCTCGAGCGCGGAGTTCG
GTGCCCCAACCGCCGAGGAAGAGGACCTGCTGGAATTCCTGGACGGCGTGCAGCCGAGCAGCCGTCTG
GCCTGCCAGCTGGTGCTGACCCCAGACATGGACACCATCACCGTGACCGTGCCGCCAGCCGACGTGTG
AAAGCTT

Ferredoxin 2 from *Rhodococcus rhodochrous* J3 (*Pst*I-*Hind*III)

GenBank accession number: MK007070

Native sequence: locus tag B9Z02_RS11995

CTGCAGAGGAGGAAAAACCCATGCCGAAGGTGTTCTACGTGCAGCCGGACGGCACCGAGCGCGTGATC
GACGGCGTGGCCGGTGACAGCGTGATGAGCACCGCCGTGCGTAACGGCGTCGCGGGTATCGTGGGTCA
GTGCGGTGGTAGCCTGAGCTGCGCGACCTGCCACGTGTATCTGGCCGCCGAGGACCAGCAGCACTTCG
ATGCCCCAAGCGAGGACGAGGACGACATGCTGGACTGCACCGCCAGCGACCGCGAGGACAGCTCGCGT
CTGAGCTGCCAGCTGATCCTGAGCGAAGGCGTGGACCTGCACGTGACCATCCCGGACGAGCAGGCCTG
AAAGCTT

Ferredoxin from *Amycolatopsis* ATCC 39116 (*Pst*I-*Hind*III)

GenBank accession number: MK007071

Native sequence: locus tag AMY39116_RS0304060

CTGCAGAGGAGGAAAAACCCATGCCGAAGATCACCTACGTGCAGCAGGACGGCAGCGCCGAGAGCTTC
GATGTGCCAGCCGGTATGAGCGTGATGGAAGCCGCCATCGAAGCCGGTGTCGGTGGCATCGTGGCCGA
GTGCGGTGGCAACGCCATCTGCGCGACCTGCCACGTGTACGTGGACCCAGGCCAGGTGGAAAAGCTGC
CGCTGCCGCAGGCCGACGAGGACGCCATGCTGGATAACACCGCCTGCCCACGTGCCGAGAACAGCCGT
CTGAGCTGCCAGATCGAGATCACCGAGGAACTGGACAGCCTGACCGTGACCGTGCCGGAAGAACAGTG
AAAGCTT
