# Supplementary material for: Identification of the two-component guaiacol demethylase system from Rhodococcus rhodochrous and expression in Pseudomonas putida EM42 for guaiacol assimilation
Source: AMB Express. 2019 Mar 11;9:34. doi: 10.1186/s13568-019-0759-8 (PMC6411806; doi:10.1186/s13568-019-0759-8)
Supplement: Supplementary file 2 — Additional file 2. Amino acid identity matrix of components from guaiacol demethylation systems. [file 13568_2019_759_MOESM2_ESM.pdf]

AMB Express

Title

Identification of the two-component guaiacol demethylase system from *Rhodococcus rhodochrous* and expression in *Pseudomonas putida* EM42 for guaiacol assimilation

Authors

Javier García-Hidalgo [a,*], Krithika Ravi [b], Lise-Lotte Kuré [a], Gunnar Lidén [b], Marie Gorwa-Grauslund [a]

[a] Division of Applied Microbiology, Department of Chemistry, Lund University, P.O. Box 124, SE-221 00 Lund, Sweden

[b] Department of Chemical Engineering, Lund University, P.O. Box 124, SE-221 00 Lund, Sweden

* Corresponding author: javier.garcia_hidalgo@tmb.lth.se Phone number: +46 462228328

Additional file S2

Amino acid identity matrix of components from guaiacol demethylation systems

Additional file S2: Clustal Omega amino acid identity matrices of the different components used in this study and homologues from other guaiacol-degrading bacteria. Accession numbers for each sequence are indicated. Identity values are expressed in percentage (%). Virtually identical proteins are marked in green.

P450 monooxygenases

	Rhodococcus rhodochrous J3 (WP_085469912.1)	*Rhodococcus opacus* 1CP (ANS30561.1)	*Amycolatopsis* sp. ATCC 39116 (WP_020419855.1)	*Rhodococcus jostii* RHA1 (WP_011595125.1)	*Rhodococcus pyridinivorans* AK37 (WP_006553158.1)
Rhodococcus rhodochrous J3 (WP_085469912.1)	-	76.4	76.7	76.4	99.0
Rhodococcus opacus 1CP (ANS30561.1)	76.4	-	78.2	99.5	75.9
Amycolatopsis sp. ATCC 39116 (WP_020419855.1)	76.7	78.2	-	78.7	76.4
Rhodococcus jostii RHA1 (WP_011595125.1)	76.4	99.5	78.7	-	75.9
Rhodococcus pyridinivorans AK37 (WP_006553158.1)	99.0	75.9	76.4	75.9	-

Redox partners

	Rhodococcus rhodochrous J3 (WP_085469913.1)	*Rhodococcus opacus* 1CP (ANS30562.1)	*Amycolatopsis* sp. ATCC 39116 (WP_020419854.1)	*Rhodococcus jostii* RHA1 (WP_011595126.1)	*Rhodococcus pyridinivorans* AK37 (WP_006553157.1)
Rhodococcus rhodochrous J3 (WP_085469913.1)	-	60.2	57.7	60.2	93.4
Rhodococcus opacus 1CP (ANS30562.1)	60.2	-	63.9	92.1	58.4
Amycolatopsis sp. ATCC 39116 (WP_020419854.1)	57.7	63.9	-	64.5	57.1
Rhodococcus jostii RHA1 (WP_011595126.1)	60.2	92.1	64.5	-	58.4
Rhodococcus pyridinivorans AK37 (WP_006553157.1)	93.4	58.4	57.1	58.4	-

Ferredoxins used in this study

	Rhodococcus rhodochrous J3 (WP_085470952)	*Rhodococcus rhodochrous* J3 (WP_085469096)	*Amycolatopsis* ATCC 39116 (WP_020416430)
Rhodococcus rhodochrous J3 (WP_085470952)	-	46.7	44.8
Rhodococcus rhodochrous J3 (WP_085469096)	46.7	-	51.4
Amycolatopsis ATCC 39116 (WP_020416430)	44.8	51.4	-
