# Supplementary material for: Identification of the two-component guaiacol demethylase system from Rhodococcus rhodochrous and expression in Pseudomonas putida EM42 for guaiacol assimilation
Source: AMB Express. 2019 Mar 11;9:34. doi: 10.1186/s13568-019-0759-8 (PMC6411806; doi:10.1186/s13568-019-0759-8)

AMB Express

Title

Identification of the two-component guaiacol demethylase system from *Rhodococcus rhodochrous* and expression in *Pseudomonas putida* EM42 for guaiacol assimilation

Authors

Javier García-Hidalgo [a,*], Krithika Ravi [b], Lise-Lotte Kuré [a], Gunnar Lidén [b], Marie Gorwa-Grauslund [a]

[a] Division of Applied Microbiology, Department of Chemistry, Lund University, P.O. Box 124, SE-221 00 Lund, Sweden

[b] Department of Chemical Engineering, Lund University, P.O. Box 124, SE-221 00 Lund, Sweden

* Corresponding author: javier.garcia_hidalgo@tmb.lth.se Phone number: +46 462228328

Additional file S3

SDS-PAGE analysis of protein expression pattern of *P. putida* EM42 strains

The protein expression pattern of the negative control strain (pSEVA424) and the five recombinant strains constructed in this study (G0, GI, GII, GIII and GIV) was analyzed by SDS-PAGE after incubation at 30°C with vigorous shaking under IPTG induction conditions. Prior to induction cells were grown in liquid LB medium until OD_{620} of 0.5, at that point 1 mM IPTG was added and incubation was extended overnight. Finally cells were harvested, washed, lysed by sonication and centrifuged to separate the soluble extract from the insoluble cell debris. 20 µg of protein from each fraction were mixed with Laemmli buffer and loaded onto a 4-20% polyacrylamide Mini-PROTEAN TGX gel.

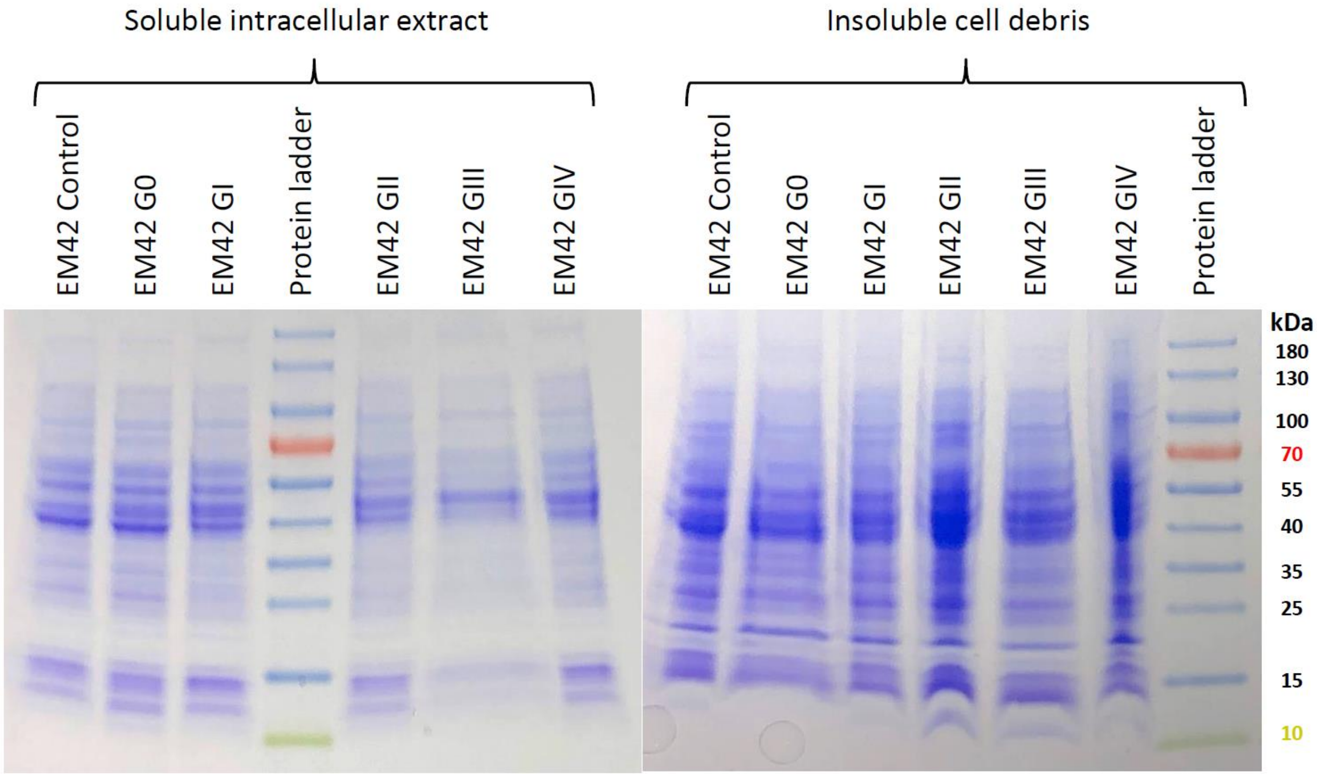

Supplement: Supplementary file 3 — Additional file 3. SDS-PAGE analysis of protein expression pattern of P. putida EM42 strains. [file 13568_2019_759_MOESM3_ESM.pdf]
