# Supplementary material for: Identification of the two-component guaiacol demethylase system from Rhodococcus rhodochrous and expression in Pseudomonas putida EM42 for guaiacol assimilation
Source: AMB Express. 2019 Mar 11;9:34. doi: 10.1186/s13568-019-0759-8 (PMC6411806; doi:10.1186/s13568-019-0759-8)
Supplement: Supplementary file 4 — Additional file 4. CFU/mL measurement of the recombinant strains used in this study. [file 13568_2019_759_MOESM4_ESM.pdf]

AMB Express

Title

Identification of the two-component guaiacol demethylase system from *Rhodococcus rhodochrous* and expression in *Pseudomonas putida* EM42 for guaiacol assimilation

Authors

Javier García-Hidalgo [a,*], Krithika Ravi [b], Lise-Lotte Kuré [a], Gunnar Lidén [b], Marie Gorwa-Grauslund [a]

[a] Division of Applied Microbiology, Department of Chemistry, Lund University, P.O. Box 124, SE-221 00 Lund, Sweden

[b] Department of Chemical Engineering, Lund University, P.O. Box 124, SE-221 00 Lund, Sweden

* Corresponding author: javier.garcia_hidalgo@tmb.lth.se   Phone number: +46 462228328

Additional file S4

CFU/mL measurement of the recombinant strains used in this study

Additional file S4: Endpoint colony forming units per mL (CFU/mL) of each strain. Average value of duplicates is shown with standard deviation. Time 0 value was taken with strain pSEVA424 with glucose only

Strain	Guaiacol only (time point 72 h)	Guaiacol + glucose (time point 23 h)
**Control (pSEVA424)**	6.33E+07 ± 6.01E+06	2.08E+09 ± 3.89E+08
**G0**	2.43E+07 ± 1.77E+06	5.45E+09 ± 2.19E+09
**GI**	4.10E+08 ± 3.54E+07	1.19E+10 ± 2.83E+08
**GII**	5.43E+08 ± 2.47E+07	7.40E+09 ± 7.78E+08
**GIII**	4.25E+08 ± 1.41E+07	7.50E+09 ± 2.12E+08
**GIV**	6.38E+08 ± 2.65E+08	6.65E+09 ± 5.66E+08
**pSEVA424 Glucose only**		1.15E+09 ± 2.29E+08
**pSEVA424 Time 0**		9.80E+07 ± 3.89E+07

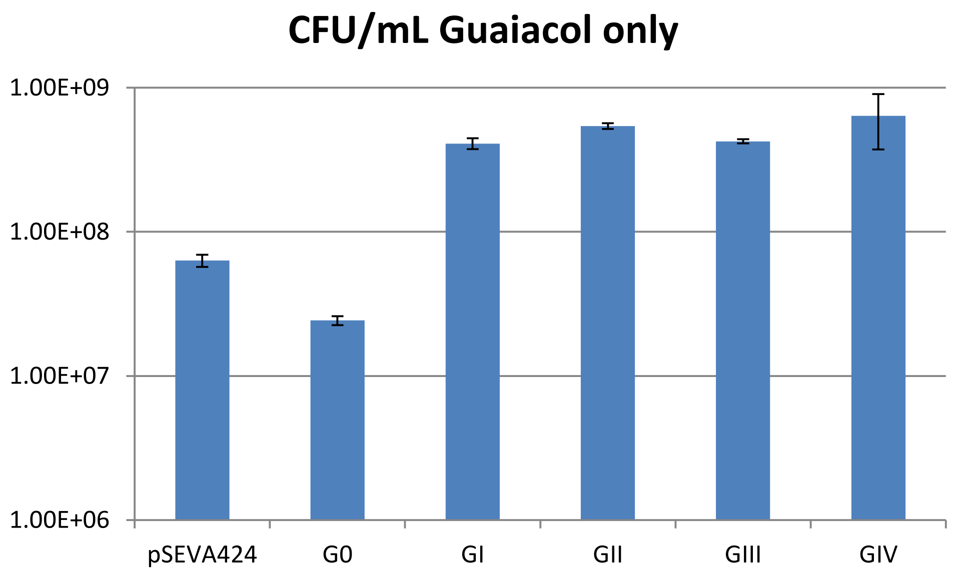

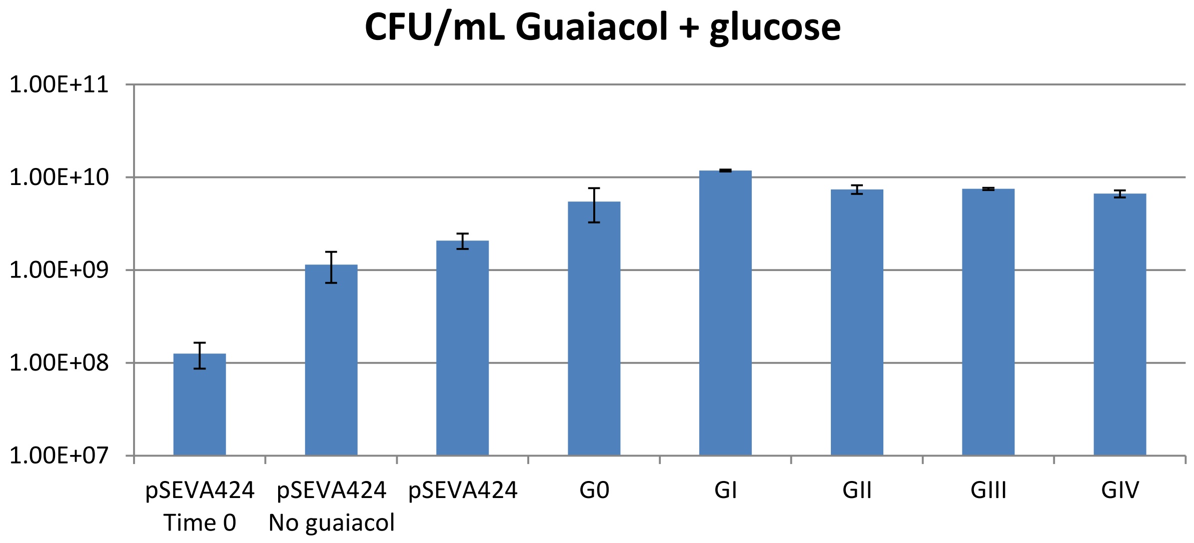
CFU/mL Guaiacol + glucose
1.00E+11
1.00E+10
1.00E+09
1.00E+08
1.00E+07
pSEVA424 Time 0
pSEVA424 No guaiacol
pSEVA424
G0
GI
GII
GIII
GIV
